# Peritoneal Endometriosis Impairs Ovarian Reserve and Increases Atresia in a Rat Model

**DOI:** 10.3390/biomedicines13020348

**Published:** 2025-02-03

**Authors:** Analía Ricci, Tatiana Bengochea, Carla Olivares, Sofía del Valle, Julieta Simone, Kristina Gemzell-Danielsson, Rosa Inés Barañao, Gabriela Meresman, Mariela Bilotas

**Affiliations:** 1 Instituto de Biología y Medicina Experimental (IBYME), Fundación del Instituto de Biología y Medicina Experimental (FIBYME), Consejo de Investigaciones Científicas y Técnicas (CONICET), Buenos Aires C1428ADN, Argentina; a.ricci@ibyme.org.ar (A.R.); bengocheats@gmail.com (T.B.); carlioliva@gmail.com (C.O.); sdelvalle782@gmail.com (S.d.V.); julietasimone1@gmail.com (J.S.); inesbaranao@gmail.com (R.I.B.); m.bilotas@ibyme.org.ar (M.B.); 2 Department of Women’s and Children’s Health, Division of Obstetrics and Gynecology, Karolinska Institutet, 17164 Solna, Sweden; kristina.gemzell@ki.se

**Keywords:** endometriosis, ovarian reserve, apoptosis

## Abstract

**Background/Objectives:** Endometriosis has a marked impact on fertility, although the mechanisms behind this relationship remain poorly understood, particularly in cases without significant anatomical distortions or in the context of ovarian endometriomas. This study aimed to investigate the effect of peritoneal endometriosis on ovarian function by assessing ovarian reserve and apoptosis. **Methods:** Peritoneal endometriosis was surgically induced in Sprague Dawley rats through the autotransplantation of uterine fragments onto the bowel mesothelium. One month post-surgery, ovarian structures were counted, follicle and corpora lutea apoptosis was evaluated by TUNEL, and apoptotic-related protein expression in ovaries was assessed by Western blot. Additionally, a co-culture system using 12Z endometriotic and KGN granulosa cell lines was utilized to evaluate gene expression by RT-qPCR. **Results:** Rats with peritoneal endometriosis exhibited a significant reduction in ovarian structures characterized by a low number of total follicles, particularly primordial, primary, preantral, and late-antral follicles. Consistently, AMH protein expression was decreased in ovaries in the presence of endometriosis. In addition, this disease led to a significant increase in late-antral follicles that were TUNEL-positive and in the number of apoptotic cells in corpora lutea, indicating higher apoptosis in endometriosis ovaries. Concomitantly, the altered expression of apoptosis-related proteins was observed, with increased procaspase 3 and decreased BCL-2 expression. In addition, KGN granulosa cells co-cultured with 12Z endometriotic cells displayed reduced KITLG mRNA expression and increased AMHR2 mRNA expression. **Conclusions:** Peritoneal endometriosis significantly impairs ovarian health by disrupting folliculogenesis, reducing ovarian reserve, and increasing apoptosis, potentially accelerating ovarian aging and contributing to infertility. These results underscore the need for further research to identify the molecular pathways involved and to develop targeted therapeutic strategies.

## 1. Introduction

Endometriosis is a complex disease characterized by the presence of endometrial-like tissue outside the uterine cavity, affecting approximately 10–15% of women of reproductive age [1]. A delayed diagnosis frequently occurs when the disease has reached an advanced stage, manifesting as chronic pelvic pain and/or infertility [2].

This condition has a particularly pronounced impact on fertility, as the prevalence of endometriosis is significantly higher among infertile women (around 30–40%) compared to the fertile population (10–15%). Surgical interventions, particularly minimally invasive laparoscopic excisions, have shown promise in alleviating pain and improving the chances of spontaneous pregnancy, with success rates reaching as high as 73% in severe cases [1,3].

The underlying mechanisms of endometriosis-related infertility are multifaceted and shaped by a range of pathophysiological processes. Severe pelvic disease can create anatomical barriers to fertility; however, the mechanisms behind infertility in women lacking significant pelvic anatomical distortions, adhesions, or the mechanical obstruction of the fallopian tubes remain poorly understood. Alternative processes need to be considered; minor adhesions, chronic intraperitoneal inflammation, luteal phase abnormalities, potential progesterone resistance, disrupted ovarian reserve/function, and disturbed folliculogenesis, are some of the possible causes of infertility linked to the disease in these patients [4].

Within the follicle, the oocyte and surrounding granulosa cells engage in a vital dialogue involving growth differentiation factor 9 (GDF-9), mammalian target of rapamycin (mTOR), and KIT ligand (KL) that ensures the proper coordination of follicular development phases and supports normal cellular function. GDF-9, expressed exclusively in oocytes beyond the primordial stage, acts as an autocrine and paracrine factor, promoting oocyte maturation and granulosa cell proliferation and differentiation. The KIT/KL pathway, activated by mTOR signaling in granulosa cells, stimulates phosphatidylinositol 3-kinase (PI3K) signaling in oocytes, awakening dormant follicles and driving oocyte growth [5,6]. Given that primordial follicles cannot be regenerated or replaced, primordial follicle activation is a critical process that regulates the maintenance and depletion of the ovarian reserve [7].

Ovarian reserve is the number and quality of the remaining primordial follicles in the ovary at any given time [8]. In clinical terms, it refers to the pool of follicles that can be stimulated, providing information about the growing population of follicles or the ovulatory potential [9]. That is why the term is often used interchangeably with other indices of ovarian function, such as antral follicle count or serum antimüllerian hormone (AMH) levels [8]. AMH, produced by granulosa cells of preantral and early-antral follicles, serves as a reliable indicator of follicle quantity and ovarian function [10].

The effect of ovarian endometriomas and their surgical removal on ovarian reserve remains a topic of ongoing debate. While many works establish a direct effect of endometrioma on the ovaries, others assert that it is its removal surgery that compromises ovarian reserve [11]. Recent research indicates that endometriomas can impair ovarian function [12], and their excision leads to a decrease in AMH levels [11,13]. Moreover, infertile women with ovarian endometriomas had lower AMH concentrations and greater prolactin levels [14]. In contrast, other background evidence suggests that endometriomas do not significantly affect ovulatory function, indicating that the relationship between endometriosis and ovarian reserve may be complex and diverse [12,15].

Despite extensive research on the relationship between endometriomas and ovarian reserve, the impact of peritoneal endometriosis on ovarian physiology remains poorly understood. To address this gap, the objective of the present work was to study the effect of peritoneal endometriosis on ovarian function by assessing ovarian reserve and apoptosis.

Our findings suggest that peritoneal endometriosis disrupts normal ovarian function by impairing folliculogenesis, reducing the primordial ovarian reserve, diminishing AMH expression, and increasing ovarian apoptosis and follicular atresia, potentially affecting fertility. Understanding the association between peritoneal endometriosis and ovarian health is crucial for improving the management of infertility associated with this prevalent form of endometriosis.

## 2. Materials and Methods

### 2.1. Animal Model

Adult (2-month-old) female Sprague Dawley rats were used in this study. Endometriosis-like lesions were induced through the transplantation of three pieces of one of the uterine horns to the bowel mesothelium using a surgical procedure adapted for rats, based on the original method described by Mc Cormack et al. [16]. Briefly, animals were deeply anesthetized with an i.p. injection of ketamine (80 mg/kg; Holliday Scott, Buenos Aires, Argentina) and xylazine (10 mg/kg; Richmond, Buenos Aires, Argentina). Rats underwent laparotomy by mid-ventral incision to expose the uterus and intestine. The distal third part of the right uterine horn was removed, opened longitudinally, and cut into square pieces measuring ~16 mm^2^. Three equal pieces of tissue were then sutured onto the serosal layer with a single 6-0 nylon suture (Supralon, Ethicon, NJ, USA) with endometrial tissue facing the serosa. Sham animals underwent the same surgical procedure, but sutures were performed on the bowels without uterine tissue.

Twenty-eight days after surgery, animals were cycled daily for 2 cycles (eight days) and euthanized afterward by CO_2_ asphyxiation in the proestrus morning. One ovary was then frozen and the other was fixed in 4% buffered formaldehyde for subsequent assays. In addition, in rats with surgically induced endometriosis, lesions were identified, counted, and measured in two perpendicular diameters using a caliper. The volume of each lesion was calculated using the following formula: V = (4/3)Πr^2^R (where r and R are the radii and r < R). Lesions were then dissected away from intestinal tissue, peritoneum, and adhesions and fixed in 4% buffered formaldehyde for two days for histological analysis. Formalin-fixed specimens of ectopic tissue were paraffin-embedded, cut into 5 µm sections, and stained with hematoxylin–eosin. Sections were examined microscopically for the presence of histological hallmarks of endometriosis.

Six rats in the Sham group and eight in the endometriosis group underwent surgery; however, one rat did not develop endometriosis and was excluded from the analysis.

### 2.2. Ovarian Morphology

The ovaries were removed and immediately fixed in 4% buffered formaldehyde for 12 h and then embedded in paraffin. Five-micrometer step sections were mounted at 50 μm intervals onto microscope slides to prevent counting the same structure twice, according to the method described by Woodruff et al. [17]. One set of slides was stained with hematoxylin–eosin to count the number of different structures per ovary section, and the others were used for apoptosis assays. Follicles were classified based on their developmental stages as follows: primordial follicles, identified by a single layer of squamous granulosa cells; primary follicles, characterized by a single layer of cuboidal granulosa cells; preantral follicles and antral follicles, distinguished by the presence or absence of an antral cavity; preovulatory follicles; and corpora lutea. Morphological characteristics of the atretic follicle include the degeneration and detachment of the granulosa cell layer from the basement membrane, the presence of pyknotic nuclei in this cell type, and oocyte degeneration [18]. The percentage of different structures was determined in 5 ovarian sections from each ovary.

### 2.3. Apoptosis Detection System

For apoptosis quantification, ovarian tissue sections were processed for terminal deoxynucleotidyl transferase (TdT)-mediated dUTP–fluorescein nick-end labeling (TUNEL) staining using the “In Situ Cell Death POD” kit (Roche, Basel, Switzerland). Sections were treated according to the manufacturer’s instructions. Briefly, sections were deparaffinized in xylene, rehydrated through graded alcohols, and permeabilized with 20 µg/mL Proteinase K (Gibco, Grand Island, NY, USA). Endogenous peroxidase was inactivated by coating the samples with 3% H_2_O_2_. Sections were rinsed with PBS and then immersed for 60 min in TdT buffer at 37 °C. Sections were incubated for 30 min with the anti-fluorescein peroxidase antibody, followed by the peroxidase substrate DAB. Finally, sections were counterstained with hematoxylin. As a negative control, some tissue samples were subjected to treatment without TdT. TUNEL-positive cells were counted using a standard light microscope by two independent observers at 400× magnification. A follicle or corpus luteum was considered TUNEL-positive when at least one apoptotic cell was present in the structure. The percentage of TUNEL-positive follicles or corpora lutea was determined for each rat by counting the total number of follicles and corpora lutea observed in a single ovarian section. Additionally, the number of TUNEL-positive cells was quantified per follicle and per corpus luteum. For corpora lutea, the percentage of TUNEL-positive cells was normalized to a constant corpus luteum area, which was calculated using the FIJI/ImageJ 1.42q software (NIH, Bethesda, MD, USA).

### 2.4. Western Blot

Ovaries were immediately frozen at −80 °C until protein extraction. Ovaries were resuspended in 500 μL of chilled lysis buffer (20 mM Tris-Cl, pH 8.0, 137 mM NaCl, 1% Nonidet P-40, and 10% glycerol), supplemented with a protease inhibitor cocktail (P8340, Sigma, Saint Louis, MO, USA), and homogenized with an Ultra-Turrax (IKA-Werke GmbH & Co, Staufen, Germany) homogenizer. The lysate was centrifuged at 13,000× *g* for 10 min at 4 °C and the pellet was discarded. Protein concentrations in the supernatant were measured by the Bradford assay [19]. Equal protein samples (30 µg) were solubilized with SDS polyacrylamide gel electrophoresis sample buffer, boiled for 5 min, and electrophoresed through a 12% SDS gel. The separated proteins were transferred to nitrocellulose membranes. Nitrocellulose membranes were blocked for 1 h in 5% low-fat powdered milk at room temperature, incubated with the primary antibodies (BCL-XS/L, Santa Cruz, Dallas, TX, USA, sc-634, 1:200; caspase 3, Santa Cruz sc-7148, 1:200; Cleaved caspase 3, Cell Signaling, Danvers, MA, USA, 9661, 1:100; BAX, Santa Cruz sc-493, 1:200; BCL-2, Santa Cruz sc-492, 1:200; AMH, Santa Cruz sc-6886, 1:200; GDF-9, Abcam, Cambridge, UK, Ab93892, 1:500; KL, Santa Cruz sc-9132, 1:200; β-actin, Abcam Ab6276, 1:2000; GAPDH, Cell Signaling #2118, 1:10,000; β-tubulin, Sigma T0198, 1:10,000), and diluted in 1% low-fat powdered milk at 4 °C. After overnight incubation, membranes were incubated with the appropriate peroxidase-conjugated secondary antibody (goat anti-rabbit IgG, Sigma A4914, 1:1000 or goat anti-mouse IgG, R&D Systems, Minneapolis, MN, USA, HAF007, 1:4000), diluted in 1% low-fat powdered milk at room temperature for 1 h, and the signal was detected by chemiluminescent substrate ECL (Pierce, Rockford, IL, USA). The protein levels were analyzed by densitometry quantification using ImageJ 1.42q software (NIH) and expressed as arbitrary units. The consistency of protein loading was evaluated using β-actin, β-tubulin, or GAPDH as loading control.

### 2.5. Co-Culture

In this study, we used the immortalized human epithelial-like endometriotic cell line 12Z [20] and the human granulosa tumor cell line KGN (RIKEN Bioresource Centre, Tsukuba, Japan). All cells were cultured in Dulbecco’s modified Eagle’s medium (DMEM), containing 10% fetal calf serum (FCS), 1% glutamine, and 1% penicillin–streptomycin.

A total of 150,000 KGN cells were seeded in 6-well culture plates, and 150,000 12Z cells were plated into transwell inserts for 6-well plates in DMEM/1%, FCS 1%, glutamine, and 1% penicillin–streptomycin. Granulosa cells were cultured alone or co-cultured with 12Z cells for 48 h.

### 2.6. Quantitative Real-Time Polymerase Chain Reaction (PCR) Analysis

KGN cell RNA isolation was performed using the Quick-RNA miniprep kit (Zymo Research, CA, USA) according to the manufacturer’s instructions. Subsequently, 10 ng of extracted RNA per sample was converted to complementary DNAs (cDNAs) using a SuperScript VILO kit (Invitrogen, Thermo Fisher Scientific, Waltham, MA, USA).

cDNAs were mixed with TaqMan gene probes (human 18S rRNA Hs99999901_s1, AMH Hs00174915_m1, AMHR2 Hs01086646_g1, MTOR Hs00234508_m1) and TaqMan master mix. The gene expressions were quantified using Step 1 real-time PCR (RT-PCR, Applied Biosystems, Thermo Fisher Scientific). Relative gene expression was evaluated using the 2^(−∆∆Ct)^ method after normalization to 18S rRNA.

Alternatively, cDNAs were mixed with the Applied Biosystems SYBR Green PCR Master Mix. To confirm the specificity of the signal obtained, melting curves were conducted in each run observed. Relative expression was calculated using ribosomal protein large P0 (RPLP0) as an endogenous control. The sequences of the oligonucleotide primers used were the following: *KITLG* (5′-AAAATCATTCAAGAGCCCAG and 5′-CCTTTCTCAGGACTTAATGTTG) and *RPLP0* (5′-ACAGGGCGACCTGGAAGTCCAACTA and 5′-AGCCCACAATTGTCTGCTCCCACA). Data were analyzed following the model of Pfaffl [21].

### 2.7. Data Analysis

Data are expressed as the means ± SEM. Representative blots and tissue sections are shown in figures. Statistical analysis was performed using an unpaired Student’s *t*-test, and outliers were identified by the Grubbs test. *p* < 0.05 was considered significant. Data were statistically analyzed using GraphPad PRISM software 6.0 (GraphPad Software Inc., Bostón, MA, USA).

## 3. Results

### 3.1. Endometriosis Alters Folliculogenesis In Vivo

**Figure 1:**
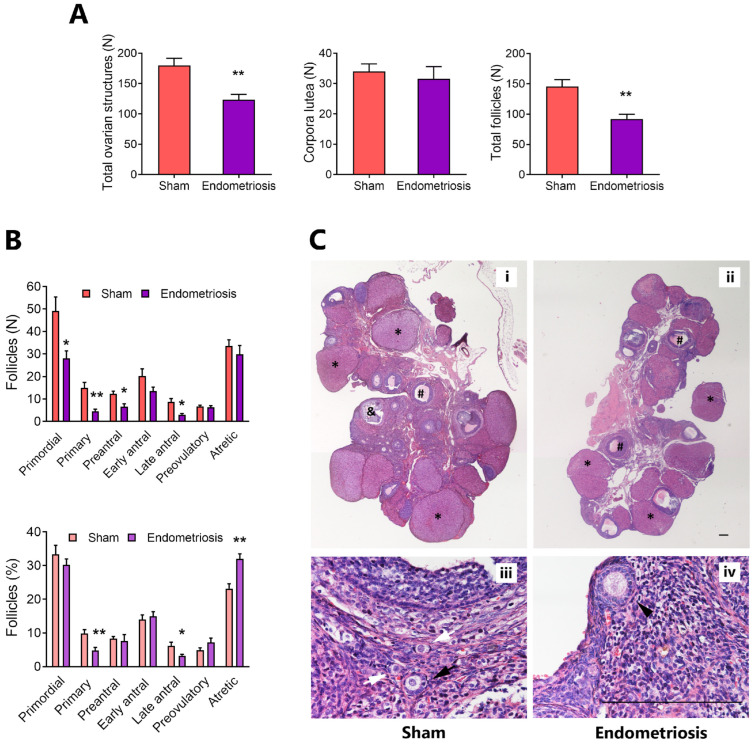
Effect of surgically induced endometriosis on folliculogenesis in vivo. (**A**): The number of follicles, corpora lutea, and total ovarian structures (i.e., follicles + corpora lutea) were assessed in ovarian sections from rats with and without endometriosis (Sham). (**B**): The number and proportion of each follicular stage were determined. (**C**): Representative micrographs show histological sections of ovaries from Sham (**i**,**iii**) and endometriosis (**ii**,**iv**) rats. Ovarian structures are indicated: corpora lutea (*), late-antral follicles (#), preovulatory follicles (&), primordial follicles (white arrows), primary follicles (black arrows), and preantral follicles (black arrowhead). Magnification 20× (**i**,**ii**) and 400× (**iii**,**iv**). Scale bar indicates 500 µm (**ii**) or 200 µm (**iv**). Results are expressed as means ± SEM. Sham: n = 6, endometriosis n = 5: statistical comparisons were performed by Student’s “*t*” test. * *p* < 0.05, ** *p* < 0.01 Sham vs. endometriosis.

To investigate the effect of endometriosis on folliculogenesis, we surgically induced peritoneal endometriosis in female adult Sprague Dawley rats, and after one month, we counted the number of follicles in each stage and the number of corpora lutea in hematoxylin–eosin-stained ovary sections (Figure 1). Rats with endometriosis showed a diminished number of total ovarian structures (i.e., follicles + corpora lutea) (*p* < 0.05 vs. Sham, Figure 1A). This decrease was related to a low number of total follicles (*p* < 0.05 vs. Sham) since no significant changes were observed in the number of corpora lutea between Sham and endometriosis rats (Figure 1A). Next, we studied the number and proportion of follicles at different stages. Data showed that rats with endometriosis have a decreased number of primordial (*p* < 0.05), primary (*p* < 0.01), preantral, and late-antral follicles (*p* < 0.05) compared to Sham rats (Figure 1B). In addition, the proportion of primary (*p* < 0.01) and late-antral follicles (*p* < 0.05) were diminished, whereas the proportion of atretic follicles was increased (*p* < 0.01) in the ovaries from endometriosis rats compared to Sham rats (Figure 1B).

### 3.2. Endometriosis Increases Ovarian Apoptosis In Vivo

**Figure 2:**
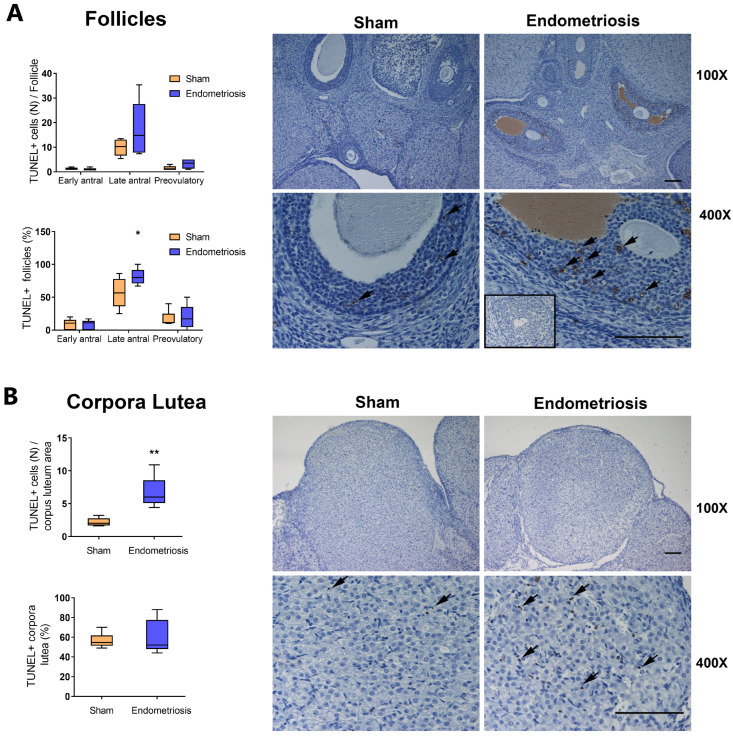
Effect of endometriosis on ovarian apoptosis in vivo. (**A**): The number of apoptotic cells per follicle and the proportion of follicles with apoptotic cells were determined by TUNEL. Micrographs of histological sections show late-antral follicles from rats with and without endometriosis (Sham). (**B**): The number of apoptotic cells per corpus luteum area and the proportion of corpora lutea with apoptotic cells were assessed by TUNEL. Micrographs of histological sections show corpora lutea from rats with and without endometriosis (Sham). Arrows indicate TUNEL-positive (TUNEL+) cells. As a negative control, an ovarian section was subjected to treatment without TdT (inset). Magnification 400× and 100×. Scale bar indicates 100 μm. Results are expressed as means ± SEM. Sham: n = 6, endometriosis: n = 6. Statistical comparisons were performed by Student’s “*t*” test. * *p* < 0.05, ** *p* < 0.01 Sham vs. endometriosis.

Since we observed an increase in the proportion of atretic follicles, we decided to evaluate apoptosis in the ovary (Figure 2). It is known that follicles are more susceptible to atresia from the early-antral stage onwards [22], so we evaluated the number of TUNEL-positive follicles (i.e., follicles that have one apoptotic cell at least) and the number of apoptotic cells per follicle in early-antral, late-antral, and preovulatory follicles from Sham and endometriosis rats.

We observed no significant changes in the number of apoptotic cells per follicle in ovaries from rats with and without endometriosis in any of the analyzed stages (Figure 2A). However, the number of late-antral follicles that were TUNEL-positive was increased in rats with endometriosis compared to Sham ones (Figure 2A, *p* < 0.05). No significant differences were seen in early-antral and preovulatory follicles (Figure 2A). In addition, rats with endometriosis showed an increase in the number of apoptotic cells per area of corpus luteum (Figure 2B, *p* < 0.01), although no changes were seen in the proportion of TUNEL-positive corpora lutea (Figure 2B).

### 3.3. Endometriosis Alters the Expression of Proteins Related to Apoptosis in the Ovary In Vivo

**Figure 3:**
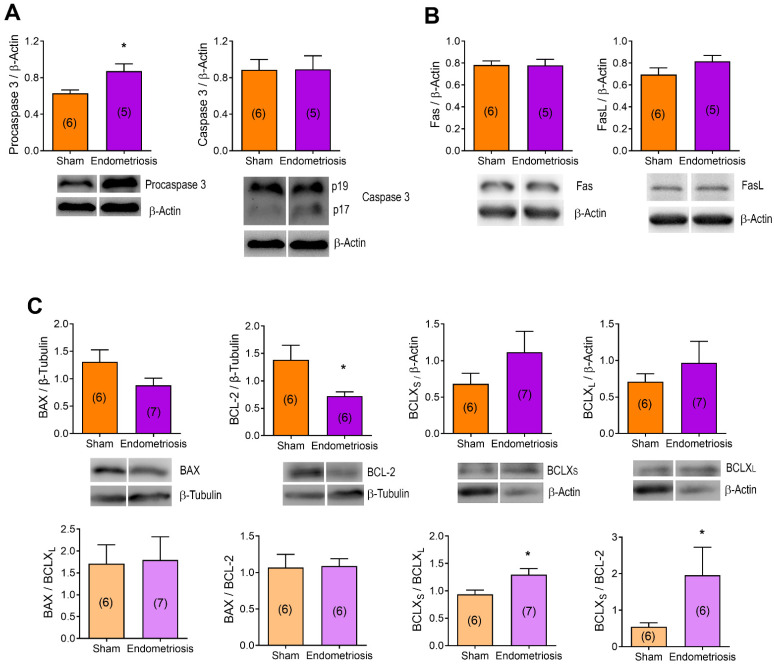
Effect of endometriosis on the expression of apoptosis-related proteins in vivo. Protein expression was evaluated by Western blot in ovarian homogenates from rats with and without endometriosis (Sham). (**A**): Procaspase 3 and caspase 3 p17 and p19 cleaved fragments; (**B**): Fas and FasL proteins (extrinsic apoptotic pathway); (**C**): BCL-2 family member proteins (intrinsic apoptotic pathway, upper panel) and proapoptotic to antiapoptotic BCL-2 family member ratios (lower panel). Results are expressed as means ± SEM. Representative blots are presented below each graph. n is expressed in parentheses in each bar. Statistical comparisons were performed by Student’s “*t*” test. * *p* < 0.05 Sham vs. endometriosis.

Hereafter, we evaluated the expression of different apoptotic-related proteins by Western blot in order to determine the pathways involved in ovarian apoptosis in rats with endometriosis (Figure 3). First, we assessed the expression of one of the most ubiquitous effector caspases, caspase 3, and its precursor, procaspase 3 (Figure 3A). Although we observed no significant changes in caspase 3 expression, the levels of procaspase 3 increased in the ovaries of rats with endometriosis (Figure 3A, *p* < 0.05 vs. Sham).

Next, we studied the extrinsic apoptotic pathway by evaluating the expression of Fas death receptor and its ligand (FasL). We saw no significant changes in the ovarian expression of these proapoptotic proteins between rats with and without endometriosis (Figure 3B). Finally, we explored the intrinsic apoptotic pathway by assessing several B-cell lymphoma 2 (BCL-2) family of proteins. The expression of the pro-survival member BCL-2 was decreased in the ovaries of rats with endometriosis (Figure 3C, *p* < 0.05 vs. Sham). Despite the presence of some tendencies, we saw no significant changes in the expression of BCL-2-associated X protein (BAX) and B-cell lymphoma-X short and long isoforms (BCL-X_S_ and BCL-X_L_) in the ovaries of rats with and without endometriosis (Figure 3C). Since the relative abundance between BCL-2 family members regulates cell fate, we assessed the ratio of proapoptotic members (BAX and BCL-X_S_) to the antiapoptotic ones (BCL-2 and BCL-X_L_). We determined that BCL-X_S_/BCL-2 and BCL-X_S_/BCL-X_L_ ratios were increased in rats with endometriosis compared to Sham rats (Figure 3C, *p* < 0.05), but there were no significant differences in BAX/BCL-2 or BAX/BCL-X_L_ ratios (Figure 3C).

### 3.4. Endometriosis Alters the Expression of mRNA and Proteins Related to Folliculogenesis In Vivo and In Vitro

**Figure 4:**
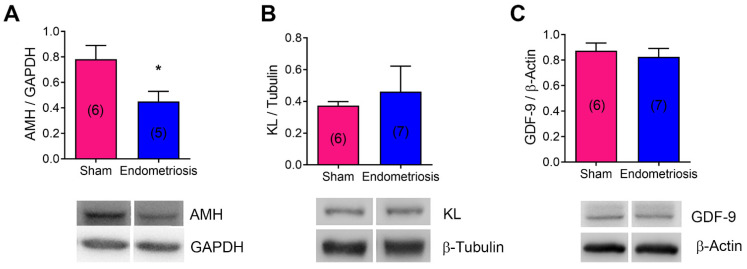
Effect of endometriosis on the expression of folliculogenesis-related proteins in vivo. Protein expression was assessed by Western blot in ovarian homogenates from rats with and without endometriosis (Sham). (**A**): AMH; (**B**): KL; (**C**): GDF-9. The upper panels show quantification, and the lower panels show representative blots. Results are expressed as means ± SEM. n is expressed in parentheses in each bar. Statistical comparisons were performed by Student’s “*t*” test. * *p* < 0.05 Sham vs. endometriosis.

**Figure 5:**
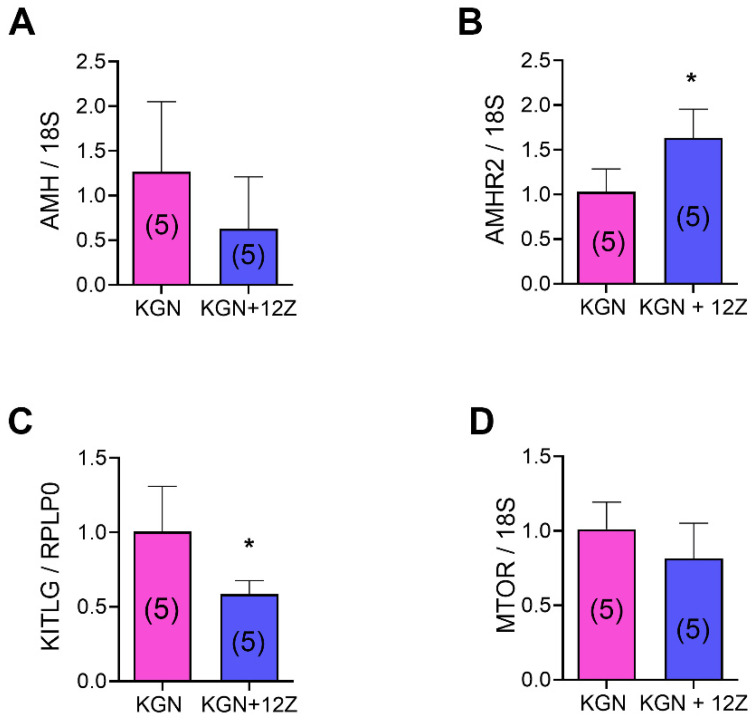
Effect of endometriotic cells on the expression of folliculogenesis-related genes in granulosa cells in vitro. The AMH (**A**), AMHR2 (**B**), KITLG (**C**), and MTOR (**D**) mRNA expressions were assessed in KGN granulosa cells by RT-qPCR. KGN cells were cultured alone (KGN) or co-cultured with 12Z endometriotic cells (KGN + 12Z) for 48 h. 18S or RPLP0 were used as housekeeping genes. Results are expressed as means ± SEM. n is expressed in parentheses in each bar. Statistical comparisons were performed by Student’s “*t*” test. * *p* < 0.05 KGN vs. KGN + 12Z.

Next, we evaluated AMH, KL, and GDF-9 protein expression in the ovaries of rats with and without endometriosis (Figure 4). Endometriosis decreased AMH protein expression (Figure 4A, *p* < 0.05 vs. Sham), although no significant changes were seen in KITLG (KL mRNA) or GDF-9 expression (Figure 4B,C). In addition, we evaluated the effect of endometriotic cell-released factors on granulosa cell protein expression in vitro. We co-cultivated the KGN granulosa cell line with the 12Z endometriotic cell line and analyzed AMH, AMH receptor 2 (AMHR2), KL, and MTOR mRNA expression in granulosa cells (Figure 5). AMHR2 mRNA expression was increased in KGN cells co-cultured with 12Z cells compared to KGN cells alone (Figure 5B, *p* < 0.05). Consistent with the result observed in vivo, KL mRNA expression was decreased in KGN cells co-cultured with 12Z cells (Figure 5C, *p* < 0.05). Although we observed no significant changes in AMH and MTOR mRNA expression in the presence of 12Z cells, there was a tendency to decrease compared to KGN cells cultured alone (Figure 5A,D).

## 4. Discussion

A substantial body of empirical evidence has established the adverse effects of endometriosis on ovarian follicles. However, the etiology and implications of this ovarian dysfunction in the context of endometriosis remain poorly understood. Ovarian impairment may stem from the intrinsic pathophysiology of endometriosis or from surgical interventions aimed at excising endometriotic lesions [12]. This is particularly evident in cases of ovarian endometriomas, where surgical procedures can directly compromise the ovarian reserve.

Moreover, some research has shown that ovarian tissue damage may occur even in the presence of ovarian endometriomas smaller than 4 cm [23]. Maintaining an altered cellular microenvironment in these cases may induce long-term cellular damage and even the malignant transformation of the surrounding normal tissue [24]. These findings suggest that endometriomas may exert a detrimental effect on ovarian tissue, even in the absence of visible morphological or molecular changes [25].

Despite these advances, little is known about how peritoneal endometriosis specifically affects ovarian follicles. Therefore, the primary aim of this study was to evaluate the impact of peritoneal endometriosis on ovarian function, pointing to ovarian reserve and apoptosis.

The ovarian reserve, composed of dormant primordial follicles, is regulated by mechanisms that continuously suppress follicular activation. The excessive activation of these follicles can lead to their premature depletion, resulting in diminished ovarian reserve. In the context of endometriosis, Kitajima et al. observed that women with ovarian endometriomas have a reduced proportion of primordial follicles and an increased proportion of growing follicles [26].

Our findings demonstrate that peritoneal endometriosis significantly reduces the total number of follicles, including primordial, primaries, preantral, and late-antral follicles. Additionally, the proportion of atretic follicles was markedly increased in the ovaries of rats with endometriosis compared to Sham ones, suggesting a deleterious effect of peritoneal endometriosis on ovarian health.

Oxidative stress plays a significant role in endometriosis, contributing to chronic inflammation and the dysregulation of immune responses. This imbalance, particularly involving reactive oxygen species (ROS), affects ovarian function, leading to impaired oocyte quality and embryo development. The activation of macrophages and mast cells further exacerbates chronic pelvic pain and inflammation [27]. Evidence has indicated the presence of systemic oxidative stress in women diagnosed with endometriosis, in addition to elevated levels of oxidative stress in their peritoneal fluid [28], which may subsequently impact the ovaries and influence folliculogenesis. Recent research indicates that oxidative stress leads to abnormal signaling pathways, accelerating ovarian aging by inducing granulosa cell apoptosis [29,30]. Apoptosis occurring within ovarian cells can lead to significant follicular atresia or regression and is regarded as a critical mechanism contributing to the phenomenon of ovarian senescence [30].

Consistently, in this work, we observed that peritoneal endometriosis led to a significant increase in late-antral follicles that were TUNEL-positive as well as in the number of TUNEL-positive cells in corpora lutea, indicating higher levels of apoptosis.

Concomitantly, the altered expression of apoptosis-related proteins was observed, with increased procaspase 3 and decreased BCL-2 expression and a major ratio of proapoptotic/antiapoptotic proteins like BCL-X_S_/BCL-2 and BCL-X_S_/BCL-XL. These findings highlight the role of the intrinsic apoptosis pathway, which is well known to be regulated by the delicate balance between proapoptotic and antiapoptotic members of the BCL-2 family. Their interactions, dictated by relative abundance and binding affinities, ultimately determine cell fate [31]. Our results strongly suggest that the intrinsic apoptotic pathway is involved in endometriosis-induced apoptosis in the ovaries of rats with endometriosis. Additionally, although no changes in Fas or FasL expression were detected, the potential contribution of other components of the extrinsic apoptotic pathway cannot be entirely excluded.

Next, we evaluated the effect of experimental peritoneal endometriosis on the expression of key molecules involved in folliculogenesis.

AMH is a well-established biomarker of ovarian reserve and a key indicator of ovarian aging [32]. Functionally, AMH plays a critical role in preserving ovarian reserve by suppressing the activation of primordial follicles. Additionally, it modulates the growth of preantral and small antral follicles by reducing their sensitivity to FSH [33].

Since AMH is only produced by granulosa cells of growing follicles, serum levels are a reflection of its ovarian expression [5]. Recently, Pedachenko et al. observed that infertile women with endometriosis, regardless of its type, had lower AMH concentrations compared with infertile women without endometriosis [14]. In addition, AMH levels in peritoneal fluid were positively correlated with serum AMH levels in both women with and without endometriosis [34]. However, as we pointed out before, most of the background in AMH and endometriosis focuses on ovarian endometriosis [35,36], leaving peritoneal endometriosis relatively understudied.

In our in vivo experimental model of peritoneal endometriosis, we observed a decrease in AMH expression, which is consistent with the mentioned decrease in the number of primordial follicles and indicates that endometriotic lesions negatively impact ovarian reserve besides not being in direct contact with them. This reduction in AMH levels may reflect impaired folliculogenesis, potentially due to disrupted granulosa cell function. Interestingly, despite the decreased AMH expression, the increased levels of AMHR2 observed in vitro may indicate an adaptive response to alterations in local signaling pathways. The elevated AMHR2 expression in granulosa cells co-cultured with 12Z endometriotic cells suggests that AMHR2, a receptor involved in follicular development signaling, may be upregulated to counterbalance the altered ovarian environment induced by endometriosis. This response is consistent with findings in other contexts, such as bovine oocyte exposure to bisphenols, where a reduction in AMH expression was accompanied by an increase in AMHR2 mRNA and protein levels [37]. Additionally, in vitro experiments showed no significant changes in AMH and MTOR mRNA expression in KGN cells co-cultured with 12Z cells, although a decreasing trend was observed compared to KGN cells cultured alone. Further studies are necessary to validate this pattern and elucidate the potential role of MTOR in mediating the adverse effects of endometriosis on ovarian reserve.

KL, a growth factor synthesized by granulosa cells, plays a key role in oogenesis and folliculogenesis [6]. Through the interaction with the KIT receptor, KL activates the PI3K/AKT signaling pathway in dormant oocytes and induces their growth [5]. A few years ago, Takeuchi et al. hypothesized that endometriomas drive excessive primordial follicle activation through the PI3K-PTEN-Akt-Foxo3 signaling pathway, a mechanism identified in both mouse models and human ovarian samples [38]. This aberrant activation can lead to a depletion of the ovarian reserve, directly affecting the patient’s fertility.

On the other hand, KL can act as an antiapoptotic factor on oocytes in primordial follicles [39] and in granulosa cells [40]. In a recent study developed in mice with the postnatal deletion of KIT, there were no defects in early follicle development. However, as mice matured, they experienced a complete loss of ovarian reserve and function, leading to infertility. Additionally, mice displayed smaller ovarian size and weight, delayed folliculogenesis, reduced AMH, and the absence of ovarian follicles [41]. At the same time, higher cleaved caspase 3 levels were observed in the granulosa cells of these ovaries, suggesting an increase in apoptosis due to the lack of the antiapoptotic signal exerted by KL [41].

In this study, KITLG mRNA expression was significantly decreased in granulosa cells co-cultured with endometriotic cells, suggesting that soluble factors released by the latter may disrupt critical processes involved in oocyte growth and follicular development and could play a role in infertility commonly associated with endometriosis.

Moreover, previous studies have highlighted the pivotal role of GDF-9 in primordial follicle activation and follicular development, as well as in stimulating the expression of KL and its receptor KIT in granulosa cells [42]. The lack of significant changes in GDF-9 and KL protein expression in the ovary in vivo suggests that endometriosis may impair ovarian apoptosis and follicular development through alternative mechanisms. These findings underscore the intricate relationship between endometriosis and ovarian function and highlight the need for further investigation into the role of KL in the infertility associated with this multifactorial disease.

In addition to the factors evaluated in our research, numerous other variables may contribute to the infertility associated with endometriosis. The roles of estrogen and progesterone in the pathophysiology of endometriosis are extensively documented [43]. Furthermore, vaginal microbiota has been recognized as a significant contributor to the pathogenesis of this condition [44]. Within this context, the estrobolome—defined as the modulation of estrogen concentrations by specific microbial genes—has emerged as a novel factor in the disease [45,46]. Notably, recent studies suggest that estradiol may play a protective role in preserving ovarian reserve through estrogen receptor β (ESR2). Both tamoxifen, a well-known estrogen receptor antagonist, and the disruption of ESR2—without affecting ESR1—result in the overactivation of primordial follicles, leading to the depletion of ovarian reserve [47,48]. This mechanism could be relevant to our model; however, further research is necessary to clarify the role of estrogen and its receptors in relation to ovarian reserve and endometriosis.

## 5. Conclusions

Our findings underscore the significant impact of peritoneal endometriosis on ovarian health, particularly through its detrimental effects on folliculogenesis and ovarian reserve. The observed reduction in follicle numbers, increased apoptosis, and downregulation of critical factors such as AMH and KL highlight the multifaceted mechanisms by which endometriosis impairs ovarian function. Additionally, the disruption of granulosa cell function and altered signaling pathways, including increased proapoptotic protein expression, suggest a complex interplay between local and systemic factors. These results emphasize the potential role of peritoneal endometriosis in accelerating ovarian aging and contributing to infertility, warranting further research to elucidate the molecular pathways involved and develop targeted therapeutic strategies.

## Data Availability

The data that support the findings of this study are available from the corresponding author, [G.M.], upon special request.
